# A Comparative Study of Oral Microbiota in Infants with Complete Cleft Lip and Palate or Cleft Soft Palate

**DOI:** 10.1155/2017/1460243

**Published:** 2017-03-14

**Authors:** Agnieszka Machorowska-Pieniążek, Anna Mertas, Małgorzata Skucha-Nowak, Marta Tanasiewicz, Tadeusz Morawiec

**Affiliations:** ^1^Department of Orthodontics, School of Medicine with the Division of Dentistry in Zabrze, Medical University of Silesia in Katowice, Traugutta Square 2, 41-800 Zabrze, Poland; ^2^Department of Microbiology and Immunology, School of Medicine with the Division of Dentistry in Zabrze, Medical University of Silesia in Katowice, Jordana 19, 41-808 Zabrze, Poland; ^3^Department of Conservative Dentistry with Endodontics, School of Medicine with the Division of Dentistry in Zabrze, Medical University of Silesia in Katowice, Akademicki Square 17, 41-902 Bytom, Poland; ^4^Department of Dental Surgery, School of Medicine with the Division of Dentistry in Zabrze, Medical University of Silesia in Katowice, Akademicki Square 17, 41-902 Bytom, Poland

## Abstract

Few reports have been published on the early microbiota in infants with various types of cleft palate. We assessed the formation of the oral microbiota in infants with complete cleft lip and palate (CLP *n* = 30) or cleft soft palate (CSP *n* = 25) in the neonatal period (T1 time) and again in the gum pad stage (T2 time). Culture swabs from the tongue, palate, and/or cleft margin at T1 and T2 were taken. We analysed the prevalence of the given bacterial species (the percentage) and the proportions in which the palate and tongue were colonised by each microorganism. At T1,* Streptococcus mitis (S. mitis)* were the most frequently detected in subjects with CLP or CSP (63% and 60%, resp.). A significantly higher frequency of methicillin-sensitive* Staphylococcus aureus* (*S. aureus* MSSA) was observed in CLP compared to the CSP group. At T2, significantly higher percentages of* S. mitis*,* S. aureus* MSSA,* Staphylococcus epidermidis*, and members of the Enterobacteriaceae family were noted in CLP infants compared to the CSP.* S. mitis* and* Streptococcus sanguinis* appeared with the greatest frequency on the tongue, whereas* Streptococcus salivarius* was predominant on the palate. The development of the microbiota in CLP subjects was characterised by a significant increase in the prevalence of pathogenic bacteria.

## 1. Introduction

The oral cavity, which remains sterile throughout prenatal development, becomes a diverse ecosystem colonised by numerous microorganisms during the first hours following delivery. The skin and mucus membranes of neonates are colonised by microbiota as a result of contact with the external environment. A significant part of the oral microbiota in the early neonatal period originates from the mother and is transient population of microorganisms consisting of intestinal bacteria (in neonates born naturally) [1]. The resident microbiota in this period depends mainly on external factors, including gestational age, mode of delivery, type of feeding, the length of hospital stay following delivery, and general condition [1–10]. The complex structure of the oral cavity, with its numerous recesses, the mucosal folds of the palate, and the invaginations of the cheeks and tongue, creates niches with different pH values, local oxygen concentrations, redox states, ionic compositions, buffer capacities, hydration, access to saliva, and mechanical interactions. These conditions are favourable for the development of a diverse ecosystem based on the interactions between bacteria and the host environment [11, 12]. The early oral microbiota occurring within several hours following delivery is composed of viridans streptococci and* Streptococcus salivarius (S. salivarius)*, which are commensals permanently colonising the oral cavity [2]. Along with other bacteria, they participate in the formation of a “colonisation cascade” that determines future indigenous microbiota [2, 5, 6].

Congenital orofacial malformation affects the structure and functions of the oral cavity, thereby significantly modifying its characteristics [13]. As a result, such malformations may exert influence on the microbiota of the environment. Orofacial clefts are the most common congenital developmental malformation of the oral cavity [14]. Neonates with complete cleft lip and palate (CLP) are characterised by the existence of communication between oral and nasal cavities extending from the upper lip and nasal vestibule to the end of the soft palate. This condition adversely affects natural sucking or even impairs the ability to swallow food [15]. Moreover, neonates and infants with orofacial cleft require specialised care to maintain proper hygiene of the incisive bone, nasal passages, and the oral cavity with special attention paid to preparation for future surgical procedures [14]. Cleft soft palate (CSP) is a less severe form of orofacial cleft with the continuity of the lips and hard palate maintained. Dysmorphia of the oral cavity in patients with this malformation affects the dorsal part of the oral and nasal cavities, which are characterised by significantly reduced communication compared to CLP [16].

Previous studies have confirmed that patients with orofacial cleft are at increased risk for the development of caries and periodontal diseases compared to noncleft children [13, 14]. Furthermore, changes in the amount and composition of oral microbiota have been reported in subjects with different types of cleft palate during deciduous or permanent dentition [17] and as the result of surgical or orthodontic treatment [18–20].

Both abnormal morphology and improper function of the oral cavity in newborns with cleft palate create a different environment from that of healthy neonates. Therefore, these abnormalities may affect oral microbiota [21]. Few reports have been published on the early microbiota in neonates and infants with various types of cleft palate.

The primary aim of the study was to compare the oral microbiota in infants with CLP and infants with CSP group. The second aim was to assess the development of the oral microbiota in subjects with complete CLP and age-matched CSP group during the neonatal period and then in the gum pad stage of the infancy period before surgery.

## 2. Materials and Methods

### 2.1. Design and Participants

This study was conducted from May 2012 to December 2014 in the Developmental Anomaly Outpatient Clinic at the Centre of Dentistry and Specialist Medicine, Medical University of Silesia in Zabrze. The study was approved by the Bioethics Committee of the Medical University of Silesia in Katowice, Poland (KNW/0022/KB1/54/12). All legal guardians of the subjects enrolled in the study provided written consent for their participation.

The study materials consisted of microbiological smears from the oral cavity mucosa collected from neonates and infants with cleft malformation who were consulted and treated at the Developmental Anomaly Outpatient Clinic of the University Centre of Dentistry and Specialised Medicine in Zabrze, Poland.

**Figure 1:**
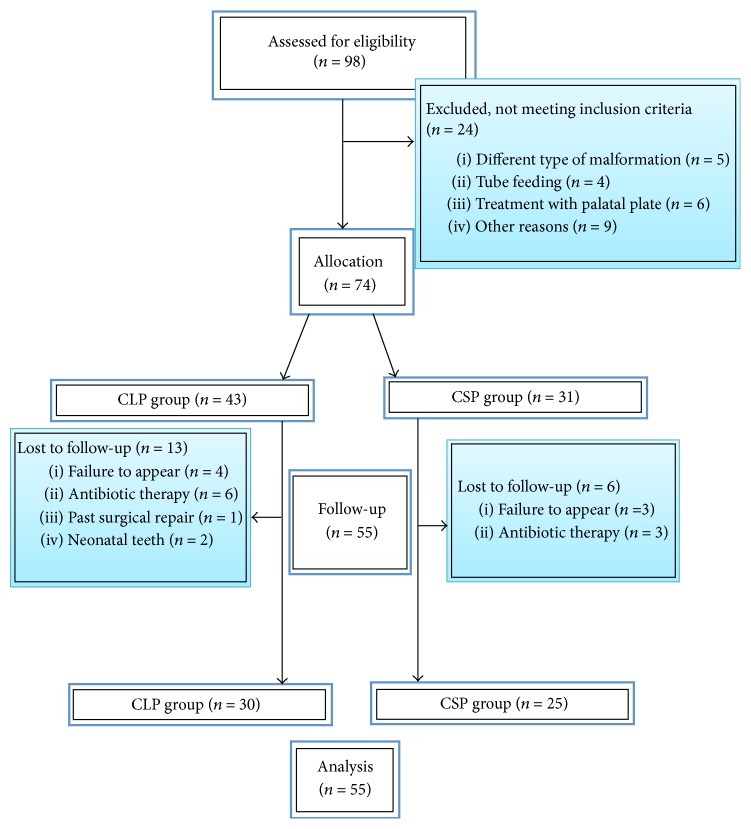
Number of subjects recruited and flow of patients within the study.

The inclusion criteria for newborns were as follows: (1) complete CLP or CSP; (2) gestational age over 37 weeks, (3) birth weight of 2,500–4,000 g, and (4) Apgar score of 9-10 at 1 min and of 10 at 5 min. The exclusion criteria were (1) the coexistence of orofacial cleft with other developmental abnormalities, (2) antibiotic therapy, (3) respiratory tract infections, (4) tube feeding, (5) treatment with palatal plate, (6) natal or neonatal teeth, (7) deciduous teeth at T2, (8) past surgical repair of cleft lip and/or palate, and (9) failure to appear for the follow-up visit between the eighth and eighteenth week of life.  Figure 1 shows a flowchart of the process that was used to screen and select the trials.

At the first visit all parents were provided with the feeding instructions that were adapted individually to the needs of each patient. All mothers were encouraged to put the child to the breast. All patients with CLP were bottle-fed with a broad or standard nipple. Eight patients with CLP were fed partially with modified milk and partially with breast milk from the bottle. The remaining newborns/neonates were given modified milk only. Two patients with CSP were breast-fed (within 3 and 6 weeks). After this period they were additionally fed with modified milk. Four neonates with CSP were bottle-fed (Haberman Feeder) with modified milk. The other patients were fed with the regular nipple and partially with modified milk and partially with breast milk from the bottle.

Feeding problems occurred in 16 patients (10 with CLP and 6 with CSP). The problems were related to the long feeding period (>40 min), choking, coughing, crying with feeding and regurgitation. In these patients a lower weight gain was observed within the first month of life by ~90–110 g per week.

The subjects were divided into two groups (Table 1). The first group consisted of 30 infants with unilateral or bilateral complete CLP. In this group, smears were obtained from palatal mucosa on the cleft margin (sample A1) and from the dorsum of the tongue (sample A2). The second group comprised 25 subjects with isolated CSP, and smears were obtained from the palatal mucosa (sample B1) and the dorsum of the tongue (sample B2). The samples were collected by rubbing the mucous membrane with a sterile cotton swab.

The smears were collected twice from the subjects of both groups. The first smear was obtained within the first or the second week of life (time T1), and the second was taken between the eighth and the eighteenth week of life (time T2), prior to cleft lip and palate repair. All samples were obtained using a sterile EUROTUBO® collection swab with Amies transport medium (DELTALAB, Rubi, Spain) and were delivered to the Department of Microbiology and Immunology in Zabrze within 1 h, where the material underwent analysis.

### 2.2. Microbiological Examination

The samples collected for microbiological investigation were smears from the palatal mucosa and smears from the dorsum of the tongue. All the studied samples were inoculated on a solid culture media from Biomerieux (Marcy l'Etoile, France): Columbia agar with 5% ram blood, MacConkey agar, Mannitol salt/Chapman agar, and Sabouraud agar. The bacteria were grown on suitable media at 37°C in aerobic conditions. Yeast fungi of the* Candida* species were multiplied on selective solid medium Sabouraud agar at a temperature of 35°C in aerobic conditions. Before identification, the studied microorganisms were cultured and isolated on solid nonselective medium Columbia agar with 5% of ram blood in order to evaluate the morphology of the pure culture, haemolytic activity, and pigmentation production. We also used related selective solid media, such as MacConkey agar for rods and Chapman agar for cocci. After isolation and further culture of each microorganism, their species were identified using the following set of reagents: Slidex Staph Plus, ID Color Catalase, Oxidase Reagent, and Api Candida (Biomerieux, Marcy l'Etoile, France), as well as STAPHYtest 24, STREPTOtest 24, ENTEROtest 24N, and NEFERMtest 24N (Erba-Lachema, Brno, Czech Republic). In the case of Gram positive, catalase negative beta-haemolytic cocci we analysed the presence of group antigens using Slidex Strepto Plus kit, and a sensitivity test to optochin was performed to clearly differentiate pneumococci from other streptococci. Species of microorganisms were identified using conventional methods, with the use of commercial test kits (STAPHYtest 24, STREPTOtest 24, ENTEROtest 24N, and NEFERMtest 24N), from among the MIKROLATEST identification kits manufactured by Erba-Lachema. The MIKROLATEST kits are a standardized micromethod system for rapid, reliable routine identification of the most clinically important bacteria and yeasts, in every case on the basis of 24 biochemical tests placed in microwells. For evaluation of identification results, we used TNW LITE 6.5 software as recommended by Erba-Lachema. Identification of the microorganism species using these reagents was performed according to the vendors' protocols.

### 2.3. Data Collection

At T1 and T2, we assessed the prevalence of the given bacterial species (the percentage) found in the oral cavities of subjects with CLP or CSP group. We also analysed the proportions in which the palate (A1, B1) and tongue (A2, B2) were colonised by each microorganism.

The intensity of bacterial growth was considered using the following scale: (1) scant growth, (2) medium growth, and (3) abundant growth. The evolution of the microbiota between T1 and T2 was assessed separately for both groups by analysing the number of patients for whom a given microorganism was detected at both timepoints or only at T1 or T2.

### 2.4. Statistics

Descriptive statistics are expressed as number and percentage and as median and interquartile range, as appropriate. The distributions of continuous variables were compared with a Mann–Whitney *U* test and proportions with the chi-square test.

The differences in the frequency of the occurrence of each bacterial species between CLP and CSP group at T1 and T2 were assessed using the chi-square test.

The McNemar test was used to compare within-group differences in the frequency of detection of single bacterial species between T1 and T2. Odds ratios (ORs) with 95% confidence intervals (CIs) were computed. All statistics were two-tailed, and the significance level was defined as* p* < 0.05. Statistical analyses were performed using Statistica v.10.

## 3. Results

### 3.1. Gum Pad Stage of the Neonatal Period (T1)

The subjects with CLP were delivered by caesarean section significantly more frequently compared to the CSP group (*p* = 0.038). The study groups were age- and birth weight-matched (Table 1).

The genus* Streptococcus* was found most frequently in both the CLP and CSP groups in the neonatal period (63%), whereas* Streptococcus mitis (S. mitis)* was the most frequently observed species (63.3% and 60.0%, resp.) (Table 2). Moreover, the frequency of methicillin-sensitive* Staphylococcus aureus* (*S. aureus* MSSA) was significantly higher in the CLP group (*p* = 0.020) than in the CSP group (Table 2).

The majority of* Streptococcus* species showed abundant growth in subjects of both the CLP and CSP groups (Table 2).

A difference in microbiota colonisation between the palate (A1, B1) and tongue (A2, B2) was observed in both groups. This change was related to the bacteria that appeared with frequencies higher than 20%.* S. mitis* and* S. salivarius* were dominant on the tongue (A2, B2), whereas* Streptococcus sanguinis (S. sanguinis)* prevailed on the palate (A1, B1). The remaining bacteria did not demonstrate significant differences in their colonisation of the palate and tongue (Table 2).

### 3.2. Gum Pad Stage of the Infancy Period (T2)

*S. salivarius* was the most frequently isolated bacterial species in both CLP and CSP patients (100% and 84%, resp.; Table 3). Furthermore, compared to the CSP group, subjects from the CLP group presented a significantly higher percentage of the following bacterial species:* S. mitis* (*p* = 0.002),* S. salivarius* (*p* = 0.022),* S. aureus* MSSA (*p* < 0.001),* Staphylococcus epidermidis (S. epidermidis)* (*p* < 0.001), and the members of the Enterobacteriaceae family, that is*, Enterobacter cloacae (E. cloacae)* (*p* = 0.007),* Klebsiella pneumoniae (K. pneumoniae)* (*p* < 0.001), and* Klebsiella oxytoca (K. oxytoca)* (*p* < 0.001) (Table 3).

The proportions in which the palate (A1, B1) and the tongue (A2, B2) were colonised by each microorganism were similar to that observed in the neonatal period.* S. mitis* and* S. salivarius* were dominant on the tongue (A2, B2), whereas* S. sanguinis* prevailed on the palate (A1, B1) (Table 3).

Moreover, infants from CLP group and from CSP group presented with* Streptococcus agalactiae (S. agalactiae)* (6.6%, 16%), and infants with CLP also presented with* Streptococcus pyogenes (S. pyogenes)* (13.3%) (Table 3).

### 3.3. Development of the Microbiota (between T1 and T2)

#### 3.3.1. CLP Group

In the CLP group, between T1 and T2, a statistically significant increase was observed in the prevalence of 9 bacterial species:* S. mitis* (*p* = 0.006),* S. sanguinis* (*p* = 0.012),* S. salivarius* (*p* < 0.001),* S. aureus* MSSA (*p* < 0.001),* S. epidermidis* (*p* < 0.001),* Neisseria* spp. (*p* = 0.007),* E. cloacae* (*p* = 0.021),* K. pneumoniae* (*p* = 0.006), and* K. oxytoca* (*p* < 0.001). Moreover, a statistically significant decrease in the percentage of* Gemella morbillorum* (*p* = 0.041) was revealed at T2. The odds ratio for* S. salivarius* in the CLP group during T2 was 22 times higher compared to T1, OR = 22 [95% CI, 2.96–16.21]. The odds ratio for* S. aureus* MSSA, OR = 16 [95% CI, 2.12–12.65], and* K. oxytoca*, OR = 18 [95% CI, 2.40–13.83], were 16 and 18 times higher, respectively, at T2 than at T1 (Table 4).

#### 3.3.2. CSP Group

A statistically significant increase in the frequency of* S. salivarius* was observed (*p* = 0.022) at T2, OR = 5.5 [95% CI, 1.219–24.814]. The frequency of the occurrence of the remaining bacteria changed insignificantly (Table 5).

## 4. Discussion

The study presents the prevalence of oral microbiota in several-day old newborns with CLP and CSP and changes in microbial population during the infant predental period prior to surgical procedure, which has not previously been described in the literature.

The prevalence of nonpathogenic commensal oral bacteria (i.e.,* S. mitis* and* S. salivarius*) was revealed in T1 in subjects from both CLP and CSP groups. Long and Swenson confirmed the ability of* S. mitis* and* S. salivarius* to adhere to oral epithelial cells in the oral cavity epithelia of 1-day-old newborns [22]. The early colonisation of the oral cavity by streptococci facilitates further colonisation by other strains and plays a crucial role in maintaining a healthy oral cavity throughout life [5, 22]. Thus, mechanisms exist to enable physiological colonisation of the mucous membrane by nonpathogenic microbiota in both CLP and CSP group patients during the neonatal period despite different local conditions related to the occurrence of cleft. An interesting finding of this study is the demonstration of the occurrence of* S. sanguinis* in tongue and palate swabs of toothless infants with CSP and CLP during T1 and T2. Arief et al., when analysing the saliva of 3-39-month-old patients with CLP, did not find* S. sanguinis*, either in the preoperative or postoperative period [21]. On the other hand, Caufield et al., in their long-term studies of saliva samples and dental plaque from infants, demonstrated that* S. sanguinis* precedes* S. mutans* colonisations and that both compete for niches on the tooth surface [23]. Other studies also show that colonisation of both species depends on tooth emergence [24, 25]. However, some authors prove that* S. sanguinis* [26] and* S. mutans* [27] may colonise the oral mucosa of predental infants and the dorsum of the tongue, which is an important ecological niche [28, 29].* S. sanguinis* is considered to be the antagonist of* S. mutans*, and early colonisation with* S. sanguinis* delays colonisation by* S. mutans*, which is considered to be a significant factor in the development of caries [23, 30]. Caufield et al. raise the question of whether this phenomenon should be used in the prevention of caries, inducing early colonisation by* S. sanguinis*, and thus delaying colonisation by* S. mutans* [23].

The distribution of* Streptococcus* species in the oral cavities of both CLP and CSP subjects demonstrated differences between the tongue and palate. The majority of* S. salivarius* and* S. mitis* strains were cultured from samples collected from the tongue (A2, B2), whereas* S. sanguinis* mainly derived from palate samples (A1, B1). This observation is consistent with the reports of other authors who confirmed a selective ability in the adherence of streptococci to oral epithelial cells [12, 31]. The majority of streptococci collected from the palate and the tongue showed abundant growth in both CLP and CSP group.

Group A *β*-haemolytic streptococci were not found in CLP or CSP group subjects in the neonatal period. However, other potentially virulent pyogenic streptococci were observed, including* Streptococcus pneumoniae (S. pneumoniae)*,* Streptococcus dysgalactiae (S. dysgalactiae),* and* Streptococcus intermedius (S. intermedius)*. Subjects with CLP presented with all members of the* Streptococcus anginosus* group (i.e.,* S. anginosus*,* S. constellatus,* and* S. intermedius*). These strains can cause acute infections, particularly in immunodeficient individuals. These inflections include brain, mouth, or liver abscesses and endocarditis, whereas* S. agalactiae* can cause bacteraemia in neonates, acute pulmonary insufficiency, and cerebrospinal meningitis [20]. In a study of the microbiota in patients with lip cleft prior to surgical intervention, Cocco et al. did not detect the presence of group A *β*-haemolytic streptococci in any of the patients. However, the researchers observed the species in only 2.3% of patients with cleft palate [32]. In contrast, Chuo and Timmons detected *β*-haemolytic streptococci in 11% of positive smears taken from patients with cleft lip and/or palate prior to surgical repair [18]. The presence of *β*-haemolytic streptococci in the oral cavity of subjects with CLP is related to postoperative complications, such as slow wound healing or the development of abscesses and fistulae [32].

*S. aureus* was detected in 40% of CLP subjects and in only 12% of CSP group subjects in the neonatal period. The difference between the study groups was statistically significant. The variation in the frequency of* S. aureus* between both groups may be related to the type of cleft and different local environmental conditions. In patients with CSP, the oral and nasal cavities form two nearly separate environments, as only the distal part of the soft palate is cleft. A different morphology is found in CLP patients, in whom the oral and nasal cavities are connected, which facilitates communication, including the transmission of mucus, food, saliva, air, and microbiota. In a study of CLP with oronasal fistulae, Tuna et al. emphasised the significance of* S. aureus* transmission from the oral to the nasal cavity in the risk of infection after the surgical treatment of patients with this malformation [19]. A slightly different opinion was expressed by Cocco et al., who questioned the pathogenicity of* S. aureus* in infants under 1 year of age [32]. Similarly, Jolleys and Savage did not observe an increased number of postoperative complications in patients with* S.aureus* detected in the preoperative period [33].

In both groups of patients, nonpathogenic streptococci were the most prevalent at T2, in the gum pad stage of the infancy period. Among nonpathogenic streptococci,* S. salivarius* and* S. mitis* were the most frequent.* S. aureus* was the most frequently detected potentially pathogenic strain and was observed in 93.3% of subjects with CLP and significantly less frequently in subjects with CSP (20%).

The formation of the microbiota in subjects with CLP proceeded differently than in CSP group subjects. An increased frequency of potential pathogens, mainly* S. aureus* and* S. epidermidis*, was observed in the CLP group. The odds ratio for these bacteria increased 16 times with development. These results may be explained by the altered anatomical conditions of the oral cavity, which disturbs self-cleaning and the flow of saliva, facilitating the retention of food in the recesses of the cleft and the nasal cavity and changing the physiological exposure of this area to oxygen and carbon dioxide [34].

The significant increase in the members of the family Enterobacteriaceae observed in subjects with CLP (i.e.,* E. cloacae*,* K. pneumoniae*, and* K. oxytoca*) may be related to the transmission of this microbiota from the external environment to the oral cavity. The frequent contact of parents' hands with the mucous membranes of the cleft lip, alveolar ridge, and incisive bone during hygiene procedures in the area of the cleft likely plays an important role in this process. The lip massage recommended by orthodontists as part of preoperative preparations may also significantly contribute to the observed changes in microbiota. Similarly, Cocco et al. indicated a high percentage of CLP subjects with Gram-negative organisms isolated preoperatively [32].

The development of the microbiota in CSP subjects was characterised by a statistically significant increase in the prevalence of* S. salivarius*, whereas the frequency of bacteria from the* Enterobacteriaceae* family decreased insignificantly. Therefore, the formation of the oral microbiota in subjects with CSP shows a tendency similar to that of healthy infants, in whom the number of the environmental Gram-negative rods decreases with age [5].

In conclusion, this study shows that (1) the development of the microbiota in subjects with CLP is accompanied by a significant increase in commensal and potentially pathogenic organisms* (S. aureus, S. epidermidis, Neisseria *spp.*, K. pneumoniae, *and* K. oxytoca)*; (2)* S. aureus* was detected in neonates with CLP significantly more frequently than in subjects without CLP. The prevalence of* S. aureus* increases significantly with the development of the child, and the odds ratio increases 16-fold. Patients with CLP are potentially at an increased risk of developing oral infectious diseases. Early oral health maintenance program in patients with CLP should be reinforced.

Further research on early oral microbiota of patients with oral clefts and its effect on later infectious diseases of the oral cavity, especially dental caries and periodontal diseases, is needed.

## Figures and Tables

**Table 1:** Descriptive statistics and statistical comparisons between CLP group and CSP group.

	CLP group (*n* = 30)	CSP group (*n* = 25)	*p* value
Age in days, median (IQR)			
At T1	7.5 (4–11)	7 (4–10)	0.931^a^
At T2	77 (66–91)	70 (63–90)	0.726^a^
Gender			
Female, *n* (%)	11 (36)	14 (56)	0.297^b^
Male, *n* (%)	19 (64)	11 (44)	0.137^b^
Mode of delivery			
Natural *n* (%)	13 (43)	16 (64)	0.139^b^
Caesarean section *n* (%)	23 (77)	9 (36)	0.038^b^
Birth weight (grams), median (IQD)	3000 (2400–3800)	3000 (2500–3800)	0.938^b^

^a^Mann–Whitney *U* test; ^b^chi-square test.

The results printed in boldface reached statistical significance (*p* < 0.05).

**Table 2:** Statistical comparison of microorganism frequency (prevalence), colonisation, and growth intensity between CLP group and CSP group at T1.

Microorganism	CLP group (*n* = 30)				*p* value^a^	CSP group (*n* = 25)			
	*F* (%)	Colonisation (%)		GI		*F* (%)	Colonisation (%)		GI
		A1	A2				B1	B2	
*Streptococcus mitis*	63.3	73	100	3	0.458	60.0	53.3	93.3	3
*Streptococcus oralis*	6.6	100	100	3	0.665	4.0	100	0	3
*Streptococcus pneumoniae*	3.3	100	100	3	0.590	4.0	100	100	3
*Streptococcus sanguinis*	20	100	50	3	0.486	28.0	85.7	57.1	3
*Streptococcus salivarius*	26.6	50	87.5	3	0.100	48.0	41.6	66.6	3
*Streptococcus vestibularis*	10	100	100	3	0.090	8.0	100	100	3
*Streptococcus bovis biovar I*	26.6	75	87.5	3	0.343	16.0	75	75	3
*Streptococcus acidominimus*	6.6	50	50	3	0.492	12.0	100	100	3
*Streptococcus dysgalactiae*	6.6	50	50	2	0.848	8.0	100	100	3
*Streptococcus uberis*	6.6	100	100	3	0.663	4.0	100	100	3
*Streptococcus anginosus*	6.6	80	100	2	0.188	—	—	—	—
*Streptococcus intermedius*	16.6	100	50	3	0.952	16.0	100	50	3
*Streptococcus constellatus*	6.6	100	50	3	0.188	—	—	—	—
*Lactobacillus *spp.	13.3	100	50	3	0.777	16.0	75	75	3
*Gemella haemolysans*	16.6	100	60	3	0.952	16.0	50	75	3
*Gemella morbillorum*	20	100	60	3	0.076	4.0	100	100	3
*Enterococcus *spp.	6.6	100	50	3	0.665	4.0	100	100	1
*Staphylococcus aureus MSSA*	40	83.3	83.3	3	**0.020**	12.0	100	75	2
*Staphylococcus aureus MRSA*	3.3	100	100	2	0.359	—	—	—	—
*Staphylococcus xylosus*	13.3	10	50	2	0.231	4.0	100	100	3
*Staphylococcus epidermidis*	33.3	20	23,3	2	0.833	36.0	88.8	88.8	2
*Staphylococcus hominis*	10	100	100	1	0.103	—	—	—	—
*Staphylococcus haemolyticus*	3.3	100	100	3	0.773	8.0	100	100	3
*Staphylococcus lugdunensis*	10	100	100	2	0.103	8.0	100	50	1
*Lactococcus lactis*	3.3	100	0	2	0.773	8.0	0	100	1
*Neisseria *spp.	16.6	100	80	2	0.099	36.0	88,8	100	2
*Moraxella *spp.	3.3	100	100	1	0.899	4.0	100	100	1
*Acinetobacter lwoffii*	6.6	100	100	1	0.665	4.0	0	100	1
*Acinetobacter baumannii*	3.3	100	100	1	0.899	4.0	100	100	3
*Enterobacter cloacae*	10	100	100	2	0.870	12.0	100	100	1
*Enterobacter kobei*	10	100	100	2	0.393	4.0	100	100	2
*Enterobacter aerogenes*	6.6	75	100	2	0.841	8.0	100	100	2
*Enterobacter asburiae*	3.3	100	100	2	0.889	4.0	100	100	2
*Serratia liquefaciens*	6.6	100	50	1	0.188	—	—	—	—
*Serratia fonticola*	10	100	33.3	2	0.465	4.0	100	100	1
*Klebsiella pneumoniae*	20	100	100	3	0.424	12.0	100	100	2
*Klebsiella oxytoca*	16.6	100	80	3	0.494	24.0	100	83,3	2
*Citrobacter *spp.	3,3	100	100	3	0.590	4.0	100	100	2
*Escherichia coli*	20	100	100	1	0.424	12.0	66.6	75	2

*F*, frequency, that is, percentage (%) of subjects from CLP or CSP group with a given microorganism; colonisation, percentage (%) of smears of a given bacterial species from the palate and/or from the tongue; A1, smears were obtained from palatal mucosa on the cleft margin in CLP subjects; A2, smears were obtained from the dorsum of the tongue in CLP subjects; B1, smears were obtained from the palatal mucosa in CSP subjects; B2, smears were obtained from the dorsum of the tongue in CSP subjects; GI, growth intensity; ^a^chi- square test; results printed in boldface have reached statistical significance (*p* < 0.05).

**Table 3:** Statistical comparison of microorganism frequency (prevalence), colonisation, and growth intensity between CLP group and CSP group at T2.

Microorganism	CLP group (*n* = 30)				*p* value^a^	CSP group (*n* = 25)			
	*F* (%)	Colonisation (%)		GI		*F* (%)	Colonisation (%)		GI
		A1	A2				B1	B2	
*Streptococcus mitis*	100	60	100	3	**0.002**	56	64	100	3
*Streptococcus oralis *	10	100	100	3	0.103	—	—	—	—
*Streptococcus pneumoniae*	13.3	100	100	3	0.777	16.0	100	100	3
*Streptococcus sanguinis*	50	100	46.6	3	0.458	40.0	100	60	3
*Streptococcus salivarius*	100	50	100	3	**0.022**	84.0	47.6	100	3
*Streptococcus vestibularis*	10.0	66.6	100	3	0.103	—	—	—	—
*Streptococcus bovis biovar I*	13.3	50	50	3	0.174	28.0	100	85,7	3
*Streptococcus acidominimus*	13,3	50	100	3	0.174	28.0	100	0.0	3
*Streptococcus agalactiae*	6.6	100	100	3	0.264	16.0	100	50	3
*Streptococcus pyogenes*	13.3	100	100	3	0.058	—	—	—	—
*Streptococcus uberis*	6.6	100	100	3	0.487	12.0	100	100	3
*Streptococcus anginosus*	13.3	50	100	2	0.058	—	—	—	—
*Streptococcus intermedius*	26.6	100	100	3	0.343	16.0	100	85.7	3
*Lactobacillus *spp.	13.3	75	100	3	0.058	—	—	—	—
*Gemella haemolysans*	26.6	62.5	100	2	0.177	12.0	100	100	3
*Enterococcus *spp.	13.3	75.0	100	3	0.885	12.0	100	66.6	3
*Staphylococcus aureus MSSA*	93.3	71.4	64.2	3	**<0.001**	20.0	80	100	3
*Staphylococcus epidermidis*	83.3	92.0	100	2	**<0.001**	28.0	71.4	57.1	3
*Staphylococcus hominis*	13.3	100	100	1	0.058	—	—	—	—
*Lactococcus lactis*	13.3	100	0.0	3	0.058	—	—	—	—
*Neisseria *spp.	53.3	56.2	93.7	3	0.695	48.0	83.3	66.6	3
*Enterobacter cloacae*	36.6	90.9	100	2	**0.007**	—	—	—	—
*Enterobacter kobei*	26.6	100	100	3	0.053	—	—	—	—
*Enterobacter aerogenes*	13.3	50	75	2	0.058	—	—	—	—
*Hafnia alvei*	3.3	100	100	2	0.359	—	—	—	—
*Klebsiella pneumoniae*	53.3	87.5	100	3	**<0.001**	—	—	—	—
*Klebsiella oxytoca*	76.6	43.4	56.5	3	**<0.001**	—	—	—	—
*Escherichia coli*	36.6	100	100	2	0.311	24.0	100	83.3	2
*Candida albicans*	6.6	100	100	2	0.190	—	—	—	—

*F*, frequency, that is, percentage (%) of subjects from CLP group or CSP group with a given microorganism; colonisation, percentage (%) of smears of a given bacterial species from the palate and/or from the tongue; A1, smears were obtained from palatal mucosa on the cleft margin in CLP subjects; A2, smears were obtained from the dorsum of the tongue in CLP subjects; B1, smears were obtained from the palatal mucosa in CSP subjects; B2, smears were obtained from the dorsum of the tongue in CSP subjects; GI, growth intensity; ^a^chi-square test; results printed in boldface have reached statistical significance (*p* < 0.05).

**Table 4:** Development of the oral microbiota between T1 and T2 in CLP subjects.

Microorganism	^†^+→+	^‡^+→−	^§^−→+	^#^−→−	*p* value^a^	OR	95% CI
	*n* (%)	*n* (%)	*n* (%)	*n* (%)			
*Streptococcus mitis*	19 (63)	0	11 (37)	0	**0.006**	NA	NA
*Streptococcus oralis*	1 (3)	0	2 (6)	27 (90)	0.479	NA	NA
*Streptococcus pneumoniae*	1 (3)	0	3 (10)	21 (70)	0.248	NA	NA
*Streptococcus sanguinis*	5 (16)	1 (3)	10 (33)	14 (47)	**0.012**	10	1.28–78.12
*Streptococcus salivarius*	7 (23)	1 (3)	22 (73)	0	**<0.001**	22	2.96–16.21
*Streptococcus vestibularis*	2 (6)	1 (3)	1 (3)	26 (87)	0.497	2	0.18–22.05
*Streptococcus bovis biovar I*	2 (6)	6 (20)	2 (6)	20 (67)	0.288	3	0.72–49.83
*Streptococcus acidominimus*	2 (6)	0	2 (6)	26 (87)	0.497	NA	NA
*Streptococcus agalactiae*	0	0	2 (6)	28 (93)	0.497	NA	NA
*Streptococcus pyogenes*	0	0	4 (13)	26 (87)	0.125	NA	NA
*Streptococcusdysgalactiae*	0	2 (6)	0	28 (93)	0.497	NA	NA
*Streptococcus uberis*	1 (3)	1 (3)	1 (3)	27 (90)	1.00	1	0.06–15.98
*Streptococcus anginosus*	1 (3)	1 (3)	2 (6)	26 (87)	1.00	2	0.18–22.05
*Streptococcus intermedius*	5 (17)	0	3 (10)	22 (73)	0.248	NA	NA
*Streptococcus constellatus*	3 (10)	1 (3)	1 (3)	25 (83)	1.00	1	0.06–15.98
*Lactobacillus *spp.	3 (10)	1 (3)	5 (17)	21 (70)	0.218	5	0.58–42.79
*Gemella haemolysans*	5 (17)	0	3 (10)	22 (73)	0.248	NA	NA
*Gemella morbillorum*	0	6 (20)	0	24 (80)	**0.041**	NA	NA
*Enterococcus *spp.	2 (6)	0	2 (6)	26 (87)	0.497	NA	NA
*Staphylococcus aureus *MSSA	12 (40)	1 (3)	16 (53)	1 (3)	**<0.001**	16	2.12–12.65
*Staphylococcus aureus *MRSA	0	1 (3)	0	29 (97)	1	NA	NA
*Staphylococcus xylosus*	0	4 (13)	4 (13)	26 (87)	0.733	1	0.25–3.99
*Staphylococcus epidermidis*	9 (30)	1 (3)	16 (53)	4 (13)	**<0.001**	16	2.12–12.65
*Staphylococcus hominis*	1 (3)	2 (6)	3 (10)	24 (80)	1	1.5	0.25–8.97
*Staphylococcus haemolyticus*	0	1 (3)	0	29 (97)	1	NA	NA
*Staphylococcus lugdunensis*	0	3 (10)	0	27 (90)	0.248	NA	NA
*Lactococcus lactis*	1 (3)	0	3 (10)	26 (87)	0.248	NA	NA
*Neisseria *spp.	3 (10)	2 (6)	13 (43)	12 (40)	**0.007**	6.5	1.46–28.80
*Moraxella *spp.	0	1 (3)	0	29 (97)	1	NA	NA
*Acinetobacter lwoffii*	0	2 (6)	0	28 (93)	0.479	NA	NA
*Acinetobacter baumannii*	0	1 (3)	0	29 (97)	1	NA	NA
*Enterobacter cloacae*	3 (10)	0	9 (30)	18 (60)	**0.021**	NA	NA
*Enterobacter kobei*	2 (6)	1 (3)	6 (20)	21 (70)	0.125	6	0.72–49.83
*Enterobacter aerogenes*	2 (6)	0	2 (6)	26 (87)	0.479	NA	NA
*Enterobacter asburiae*	0	1 (3)	0	29 (97)	1	NA	NA
*Serratia liquefaciens*	0	2 (6)	0	28 (93)	0.479	NA	NA
*Serratia fonticola*	0	3 (10)	0	27 (90)	0.248	NA	NA
*Klebsiella pneumoniae*	5 (17)	1 (3)	11 (37)	13 (43)	**0.006**	11	1.42–85.20
*Klebsiella oxytoca*	4 (13)	1 (3)	18 (60)	7 (23)	**<0.001**	18	2.40–13.83
*Citrobacter *spp.	0	1 (3)	0	29 (97)	1	NA	NA
*Escherichia coli*	1 (3)	5 (17)	10 (33)	14 (47)	0.301	2	0.68–5.85

^†^ Number (*n*) and percentage (%) of patients with the bacterial species present at both T1 and T2.

^‡^ Number (*n*) and percentage (%) of patients with the bacterial species present at T1 but not at T2;

^§^Number (*n*) and percentage (%) of patients without the bacterial species present at T1 but with it at T2.

^#^Number (*n*) and percentage (%) of patients without the bacterial species present at T1 or T2.

^a^McNemar Test; OR, odds ratio; 95% CI, confidence interval of 95%; NA, not applicable.

Results in boldface have reached statistical significance (*p* < 0.05).

**Table 5:** Development of the oral microbiota between T1 and T2 in CSP subjects.

Microorganism	^†^+→+	^‡^+→−	^§^−→+	^#^−→−	*p* value^a^	OR	95% CI
	*n* (%)	*n* (%)	*n* (%)	*n* (%)			
*Streptococcus mitis*	13 (52)	2 (8)	1 (4)	9 (36)	1.00	NA	NA
*Streptococcus oralis *	0	1 (4)	0	24 (96)	1.00	NA	NA
*Streptococcus pneumoniae*	1 (4)	0	3 (12)	21 (84)	0.248	NA	NA
*Streptococcus sanguinis*	4 (16)	3 (12)	6 (24)	12 (48)	0.507	2	0.50–7.99
*Streptococcus salivarius*	10 (40)	2 (8)	11 (44)	2 (8)	**0.022**	5.5	1.21–24.81
*Streptococcus vestibularis*	0	2 (8)	0	23 (92)	0.479	NA	NA
*Streptococcus bovis biovar I*	1 (4)	3 (12)	6 (24)	15 (60)	0.507	2	0.50–7.99
*Streptococcus acidominimus*	1 (4)	2 (8)	6 (24)	16 (64)	0.289	3	0.60–14.86
*Streptococcus agalactiae*	0	0	4 (16)	21 (84)	0.133	NA	NA
*Streptococcus pyogenes*	0	0	0	0	1.00	NA	NA
*Streptococcus dysgalactiae*	0	2 (8)	0	23 (92)	0.479	NA	NA
*Streptococcus uberis*	0	1 (4)	3 (12)	21 (84)	0.617	3	0.31–28.84
*Streptococcus anginosus*	0	0	0	0	1.00	NA	NA
*Streptococcus intermedius*	2 (8)	2 (8)	2 (8)	19 (76)	0.617	1	0.14–7.09
*Streptococcus constellatus*	0	0	0	0	1.00	NA	NA
*Lactobacillus *spp.	0	4 (16)	3 (12)	18 (72)	0.723	1.333	0.29–5.95
*Gemella haemolysans*	3 (12)	1 (4)	0	21 (84)	1.00	NA	NA
*Gemella morbillorum*	0	4 (16)	0	21 (84)	0.125	NA	NA
*Enterococcus *spp.	0	1 (4)	3 (12)	21 (84)	0.627	3	0.31–28.84
*Staphylococcus aureus *MSSA	1 (4)	2 (8)	4 (16)	18 (72)	0.687	2	0.36–10.91
*Staphylococcus xylosus*	0	1 (4)	0	24 (96)	1.00	NA	NA
*Staphylococcus epidermidis*	5 (20)	4 (16)	2 (8)	14 (56)	0.687	2	0.36–10.91
*Staphylococcus hominis*	0	0	0	0	1.00	NA	NA
*Staphylococcus haemolyticus*	0	2 (8)	0	23 (92)	0.479	NA	NA
*Staphylococcus lugdunensis*	0	3 (12)	0	22 (88)	0.248	NA	NA
*Lactococcus lactis*	0	1 (4)	2 (8)	22 (88)	1.00	2	0.18–22.05
*Neisseria *spp.	9 (36)	0	3 (12)	13 (52)	0.248	NA	NA
*Moraxella *spp.	0	1 (4)	0	24 (96)	1.00	NA	NA
*Acinetobacter lwoffii*	0	0	0	0	1.00	NA	NA
*Acinetobacter baumannii*	0	0	0	0	1.00	NA	NA
*Enterobacter cloacae*	0	3 (12)	0	22 (88)	0.248	NA	NA
*Enterobacter kobei*	0	0	0	0	1.00	NA	NA
*Enterobacter aerogenes*	0	2 (8)	0	23 (88)	0.479	NA	NA
*Enterobacter asburiae*	0	0	0	0	1.00	NA	NA
*Hafnia alvei*	0	1 (4)	0	24 (96)	1.00	NA	NA
*Serratia liquefaciens*	0	0	0	0	1.00	NA	NA
*Serratia fonticola*	0	0	0	0	1.00	NA	NA
*Klebsiella pneumoniae*	0	12 (48)	0	13 (52)	1.00	NA	NA
*Klebsiella oxytoca*	0	6 (24)	0	19	0.312	NA	NA
*Citrobacter *spp.	0	1 (4)	0	24 (96)	1.00	NA	NA
*Escherichia coli*	1 (4)	2 (8)	5 (20)	17 (68)	0.453	2.5	0.48–12.88
*Candida albicans*	0	1 (4)	0	24 (96)	1.00	NA	NA

^†^Number (*n*) and percentage (%) of patients with the bacterial species present at both T1 and T2.

^‡^Number (*n*) and percentage (%) of patients with the bacterial species present at T1 but not at T2.

^§^Number (*n*) and percentage (%) of patients without the bacterial species present at T1 but with it at T2.

^#^Number (*n*) and percentage (%) of patients without the bacterial species present at T1 or T2.

^a^McNemar Test; OR, odds ratio; 95% CI, confidence interval of 95%; NA, not applicable.

Results in boldface have reached statistical significance (*p* < 0.05).
